# High Rate of Mobilization for *bla*_CTX-M_s

**DOI:** 10.3201/eid1403.070405

**Published:** 2008-03

**Authors:** Miriam Barlow, Rebecca A. Reik, Stephen D. Jacobs, Mónica Medina, Matthew P. Meyer, John E. McGowan, Fred C. Tenover

**Affiliations:** * University of California, Merced, California, USA; † Emory University, Atlanta, Georgia, USA; ‡ Centers for Disease Control and Prevention, Atlanta, Georgia, USA

**Keywords:** CTX-M, phylogenetic analysis, plasmid, mobilization, beta-lactamase, evolution, antimicrobial resistance, test for selection, Bayesian inference, class A, research

## Abstract

We constructed a phylogenetic analysis of class A β-lactamases and found that the *bla*_CTX-M_*s* have been mobilized to plasmids ≈10 times more frequently than other class A β-lactamases. We also found that the *bla*_CTX-M_s are descended from a common ancestor that was incorporated in ancient times into the chromosome of the ancestor of *Kluyvera* species through horizontal transfer. Considerable sequence divergence has occurred among the descendents of that ancestral gene sequence since that gene was inserted. That divergence has mainly occurred in the presence of purifying selection, which indicates a slow rate of evolution for *bla*_CTX-M_s in the pre–antimicrobial drug era.

## Abstract 2

The *bla*_CTX-M_s have been mobilized to plasmids more frequently than other class A β-lactamases.

Antimicrobial drug–sensitive bacteria become resistant to antimicrobial drugs through a variety of mechanisms, such as chromosomal mutations that up-regulate the expression of antibiotic-resistance genes, DNA uptake through transformation, or the process of conjugation. The ability of plasmids to evolve independently of their hosts has allowed numerous resistance genes from diverse species of bacteria to assemble within single plasmids and spread into a wide variety of organisms (*1*). The mobilization of a chromosomal resistance gene to a plasmid is an important event because the mobilized gene is now capable of spreading widely throughout diverse species of bacteria and because the fitness advantage that a plasmid confers generally increases as it acquires more resistance genes (*1*).

The class A β-lactamases have been the most frequently encountered plasmidic resistance genes. Class A β-lactamases from the TEM group have occurred at a particularly high frequency; in many surveillance studies, they have been identified as the resistance determinants most frequently encountered (*2*–*9*). The first *bla*_TEM_ allele, *bla*_TEM-1_, is a plasmidic allele that was first isolated in 1963 (*10*,*11*). Currently, ≈160 different plasmidic alleles encode unique TEM β-lactamase enzymes (www.lahey.org/Studies), and all are descended from a single plasmidic ancestor, *bla*_TEM-1_ (*12*).

The SHVs are another group of class A β-lactamases that have been frequently observed in surveillance studies. As with the TEMs, numerous alleles encode unique SHV enzymes (≈105), and the SHVs are all descended from a single ancestor (*13*). The first *bla*_SHV_ allele was detected in 1974 (*10*,*14*). Unlike *bla*_TEM_s, the *bla*_SHV_s are present in the chromosome of nearly all *Klebsiella pneumoniae* isolates belonging to the KP1 group. Evidence suggests that bla_SHV_s have been chromosomally located since the pre–antimicrobial drug era (*15*), and they may have been mobilized to plasmids up to 4 times, although the sequence divergence among them is insufficient to clearly resolve the independent mobilizations of the *bla*_SHV_s.

The CTX-Ms are another group of class A β-lactamases that are located on plasmids and that have been of particular clinical interest because they are rapidly spreading through clinical populations of bacteria. The first plasmidic *bla*_CTX-M_ observed in human-associated clinical populations was isolated in 1989 (*16**,**17*). Unlike the usual pattern of class A β-lactamase mobilizations in which the plasmidic alleles are all descended from a single common plasmidic ancestor, evidence shows that CTX-Ms have been mobilized numerous times from the chromosomes of *Kluyvera* (*16*,*18*–*21*). Because *Kluyvera* chromosomal genes have been found that exactly match the sequence of plasmidic CTX-Ms (*18*), many of the mobilizations have likely occurred recently. To investigate the mobilization of the *bla*_CTX-M_s to plasmids, we generated a phylogenetic analysis of the CTX-Ms that included a representative sampling of other class A β-lactamases.

## Methods

### BLAST Search

*bla*_CTX-M_ and *bla*_CTX-M_ homologue DNA sequences were identified with a TBLASTN (www.ncbi.nlm.nih.gov/blast) (*22*,*23*) search of the nonredundant National Center for Biotechnology Information (NCBI) sequence database and the completed microbial genomes database by using characterized *bla*_CTX-M_s as query sequences (*bla*_CTX-M1_ and *bla*_CTX-M2_). The BLAST search of the completed microbial genomes identified positive matches for organisms that contain *bla*_CTX-M_ homologs. The BLAST search of completed genomes also showed which microbes have no close *bla*_CTX-M_ homologue, and thus enabled horizontal transfer events to be identified.

### Alignment

The protein sequences of the *bla*_CTX-M_s and their homologs were aligned with ClustalX 1.8 (*24*) by using the Gonet 250 similarity matrix with a gap-opening penalty of 35 and a gap-extension penalty of 0.75 for the pairwise alignment stage, and a gap-opening penalty of 15 and a gap extension penalty of 0.3 for the multiple alignment stage. The corresponding DNA coding sequences were aligned by introducing triplet gaps between codons corresponding to gaps in the aligned protein sequences with the CodonAlign program. (CodonAlign for Macintosh and for PC [Windows] computers and source code that can be compiled for other platforms are available at no charge from http://sinauer.com/hall.)

### Estimation of Positive Selection

**Figure 1:**
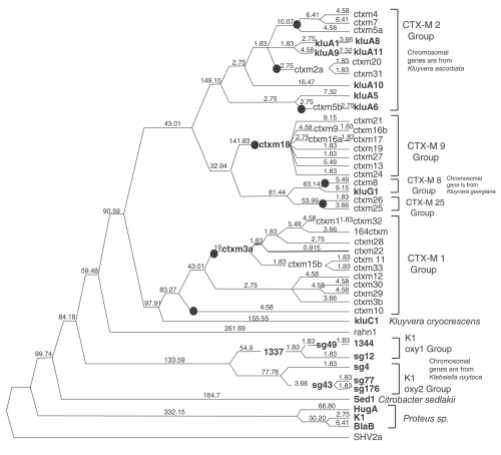
Phylogenetic analysis of *bla*_CTX-M_s. This tree was calculated by Bayesian inference. Number of mutations occurring along each branch are given along the length of the branch. Black dots represent mobilizations. **Boldface** indicates chromosomal genes. CTX-M-14 and 3a exist as both unmobilized chromosomal genes and plasmid-borne CTX-M alleles.

Estimation of the nonsynonymous (d_N_) and synonymous (d_S_) substitution rates is an important means of understanding mechanisms of molecular evolution. A d_N_/d_S_ ratio >1 is taken as evidence of positive selection, whereas a d_N_/d_S_ ratio <1 is taken as evidence of purifying selection (*25*). The Codeml program of the PAML package (available from http://envgen.nox.ac.uk/bioinformatics/docs/codeml.html) (*25*) was used to estimate d_N_/d_S_ ratios in the phylogenetic analysis shown in Figure 1. The values were calculated by using model 1 in the program, and default parameters were used for the execution of the program.

### Phylogenetic Reconstruction

Phylogenies were constructed by the Bayesian method, as implemented by the program MrBayes (*26*) (available at no charge from www.mrbayes.net). The evolutionary model used was the General Time Reversible model (*27*). Because evolutionary rates are not homogeneous for every site in a gene, among-site variation in evolutionary rate was estimated separately for first, second, and third positions of sites within codons. Four chains, with a “temperature” of 0.2 for the heated chains, were run for each tree. Trees were sampled every 100 generations. A total of 10 million generations were run with a burn-in of 5,000 trees. The length of each burn-in was set at a value that exceeded twice the number of trees required for convergence upon a stable likelihood value. Because the consensus trees calculated by MrBayes do not include the posterior probabilities of the clades, each entire set of trees was imported into PAUP* (*28*) and the same trees used by MrBayes to calculate a consensus were used to calculate a 50% majority rule consensus in PAUP* (*28*). The resulting tree shows the posterior probabilities of the clades, i.e., the percentage of time that those taxa are included in the clade. The consensus trees calculated by MrBayes were imported into PAUP* for the purposes of displaying and printing the tree.

## Results

### Ancient Horizontal Transfer of *bla*_CTX-M_ Ancestor

The NCBI genomes database (www.ncbi.nlm.nih.gov:80/entrez/query.fcgi?db=genome) currently contains the completed genomic sequences of 139 eubacterial organisms. Because *Kluyvera* are members of the *Enterobacteriaceae* group of the gamma subdivision of Proteobacteria, the genomes of other members of the gamma subdivision, and especially the chromosomes of *Enterobacteriaceae*, were of the greatest interest. BLAST searches of these microbial genomes show that among the complete genomic sequences available for 9 species of *Enterobacteriaceae (Escherichia coli*, *Salmonella typhimrium* LT2, *S. enterica*, *Shigella flexneri*, *Photorhabdus luminescens, Buchnera aphidicola, Candidatus Blochmannia floridanus, Wigglesworthia glossinidia,* and *Yersinia pestis*) none contain chromosomal homologs of the *bla*_CTX-M_s that are detectable through sequence comparison. BLAST searches similarly show that many non-*Enterobacteriaceae* members of the gamma subdivision of Proteobacteria, *Shewanella oneidensis, Haemophilus ducreyi, H. influenzae, Pseudomonas aeruginosa, P. putida, P. syringae, Vibrio cholerae, V. parahemolyticus, V. vulnificus, Xanthomonas axonopodis, X. campestris,* and *Xylella fastidiosa,* also do not contain chromosomal homologs of the *bla*_CTX-MS_. However, BLAST searches of the translated nonredundant nucleotide database revealed that *Kluyvera* species, *Citrobacter sedlakii*, and *Klebsiella oxytoca* contain close chromosomal homologs of the *bla*_CTX-MS_.

These results show that the *bla*_CTX-M_ homologs originally came into the chromosomes of *K. oxytoca, Kluyverra* species, and *C. sedlakii* by horizontal transfer because most species of gamma Proteobacteria do not contain *bla*_CTX-M_ homologs. If *bla*_CTX-M_ homologs were vertically transmitted into the species that contain them, numerous deletions would be required to explain absence of those homologs in the majority of gamma Proteobacteria. However, only 3 horizontal insertions are required to explain the presence of *bla*_CTX-M_ homologs in the chromosomes of *K. oxytoca*, *Citrobacter* species. Because fewer insertions than deletions are required to explain these data, insertion of *bla*_CTX-M_ homologs into the chromosomes of those bacteria that contain them is the most likely explanation for their current distribution.

### Divergence of the *bla***_CTX-M_**s

The GenBank DNA and protein accession numbers of the sequences included in this analysis are shown in the online Appendix Table (available from www.cdc.gov/EID/content/14/3/423-appT.htm), along with the organism in which the gene exists and whether the gene was located on a plasmid or a chromosome. The results of our phylogenetic analysis are presented in Figure 1. The groupings of *bla*_CTX-M_s on our phylogenetic tree agree with the dendrogram published by Bonnet in a recent review (*16*); for purposes of clarity, we will use the same group names used in that review, as shown in Figure 1.

The phylogenetic analysis shows that the *bla*_CTX-M_s represent a fairly divergent group of β-lactamase genes descended from a common ancestor. The genes encoding the CTX-M-1 and CTX-M-2 groups are separated by over 400 mutations, which indicates considerable diversification of the *bla*_CTX-M_s. The average distance separating the *bla*_CTX-M_s from their most recent common ancestor is 226.2-nt ± 22.8-nt mutations, which indicates that the rates of evolution among the *bla*_CTX-M_s have been similar.

Positive selection testing within the phylogenetic analysis shows that positive selection has occurred throughout the evolutionary history of the class A β-lactamases. More positive selection appears to exist at branches deep within the tree than along more recent branches. The branches during which most of the divergence of the *bla*_CTX-M_s occurred are characterized by purifying selection. The detection of purifying selection suggests a slow evolutionary rate and that the *bla*_CTX-M_s diverged in ancient times. More recent evolution of the *bla*_CTX-M_s likely can be characterized by intense positive selection, but the branches at the tips are still too short to obtain reliable dn/ds ratios.

### Mobilization of *bla*_CTX-MS_ to Plasmids

The *bla*_CTX-M_s have been mobilized from the chromosomes of various *Kluyvera* species to plasmids at least 8 times since they diverged from their most recent common ancestor as indicated in Figure 1. The alleles in the CTX-M-2 group have been mobilized from the chromosome of *Kluyvera ascorbata* at least twice (*29*). The alleles in the CTX-M-9 group have been mobilized once from the chromosome of *Kluyvera* g*eorgiana* (*30*). The alleles from the CTX-M-8 group were mobilized once from the chromosome of *K. georgiana* (*20*). The CTX-M-25 group has been mobilized once, although the species from which it originates has not yet been determined. The alleles in the CTX-M-1 group have been mobilized at least 3 times (*17**,**18**,**31*), and one of those mobilizations has been from the chromosome of *K. ascorbata*. When compared with the *bla*_TEM_s, which have been mobilized once, and the *bla*_SHV_s, which have been reported to have been mobilized 2–4 times (*32*), the number of mobilization events that have occurred among the *bla*_CTX-M_s is atypically high.

**Figure 2:**
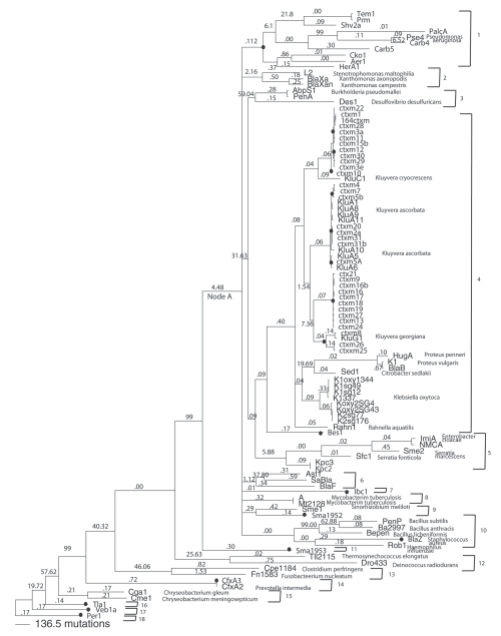
Phylogenetic analysis of class A β-lactamases calculated by Bayesian inference. Number of mutations occurring along each branch are represented visually by the lengths of the branches. d_N_ /dS ratios for all branches except the tips are given along the lengths of the branches. **Boldface** indicates plasmidic genes. Black dots indicate mobilizations to plasmids. Numbered brackets indicate monophyletic divisions within the tree. *d_N_, nonsynonymous substitution rate; d_S_, synonoymous substitution rate.

To compare the number of mobilizations that have occurred in the CTX-M group with those that have occurred in the rest of the class A β-lactamases, we constructed a phylogenetic analysis of class A alleles that spans the breadth of this group and that contains representatives of all groups of class A alleles known to the authors (Figure 2). Among all of the class A β-lactamases, including the *bla*_CTX-M_s_,_ only 22 mobilizations to plasmids were found. To quantitatively compare the numbers of times that CTX-Ms have been mobilized to plasmids with the number of times that other class A β-lactamases have been mobilized to plasmids, the total number of mutations that have occurred within the *bla*_CTX-M_ clade were summed and divided by the number of mobilizations that have occurred in that region of the phylogenetic analysis. Among the *bla*_CTX-M_s, the ratio of mobilizations to mutations is 1 mobilization per 191 mutations. Among the remainder of the tree when the *bla*_CTX-M_ clade is excluded from the analysis, 14 mobilizations occur with the ratio of mobilizations to mutations being 1 mobilization per 2,471 mutations. When the complete phylogenetic analysis is considered, 1 mobilization occurs per 1,870 mutations. By that comparison, the mobilization of the *bla*_CTX-M_ genes to plasmids has occurred at an unusually high rate. This result is unlikely to be an artifact of sampling bias or clinical interest because other class A β-lactamases have been intently studied for a longer period than the *bla*_CTX-M_s. If any bias exists in the data, it would be the undersampling of *bla*_CTX-M_ mobilizations relative to other class A β-lactamases.

Because nearly one half of the mobilizations that have occurred in the class A phylogenetic analysis have occurred among the *bla*_CTX-MS_, it seemed reasonable to conclude that the circumstances associated with the mobilizations of the *bla*_CTX-MS_ may differ from the circumstances associated with the mobilizations of other class A β-lactamases. To rule out any effect that varying intensities of selection or varying evolutionary rates might have on mobilizations to plasmids, we divided the phylogenetic analysis into several monophyletic groups for further analysis. Within the class A β-lactamase phylogenetic tree (Figure 2) are several monophyletic clades that descended from a single ancestor (node A). Each of the monophyletic clades that descended from node A were considered separately during positive selection testing except for the monophyletic clade that contains the *bla*_CTX-M_s; it was divided into 2 separate clades so that a clade containing only the *bla*_CTX-M_s and their closest relatives could be considered. Monophyletic clades that diverged before the point represented at node A were also examined individually.

The dn/ds ratios were computed for each clade (Table), and a correlation coefficient for mobilizations and dn/ds ratios of 0.21 (p = 0.40) was calculated. The average distance of each clade from the root of the tree was also computed (Table), and the correlation coefficient for mobilizations and average distance from the root is 0.21 (p = 0.41). The nonsignificant p values yielded by those results mean that the unusually high number of mobilizations among the blaCTX-Ms are probably not an artifact caused by positive selection or evolutionary rate.

**Table:** Average distances from root and dn/ds ratios of monophyletic clades

Clade	Average distance from root	d_N_/d_S_ ratio*	Mobilizations
1	3140.865	0.0935	5
2	2534.35	0.4535	0
3	2538.9	0.2446	0
4	2819.37634	0.0929	9
5	3139.5	0.0426	0
6	2575.3	0.5024	0
7	2975.7	0.1019	1
8	2443.35	0.2618	0
9	2532.075	0.1969	1
10	3267.81	0.0338	0
11	2429.7	0.0118	1
12	2811.9	0.016	0
13	2218.125	1.1595	0
14	1747.2	0.9331	1
15	873.6	0.2056	0
16	518.7	0.1069	1
17	559.65	0.1595	1
18	778.05	0.2496	0

*d_N_, nonsynonymous substitution rate; d_S_, synonoymous substitution rate.

Most of the mobilizations of the *bla*_CTX-M_s have occurred in recent years because genes that are identical (*bla*_CTX-M3a_ [*18*] and *bla*_CTX-M-18_ [*19*]) or nearly identical to the ancestors of plasmidic clades (Figure 1) have been found in the chromosomes of *Kluyvera* species, whereas many of the other plasmidic class A β-lactamases have been mobilized much longer, perhaps even since ancient times. In many cases, no chromosomal ancestor is identified and the plasmidic resistance genes are not closely related to the chromosomal resistance genes of any identified groups of bacteria.

## Discussion

Although the use of antimicrobial agents generally has enhanced the spread of antimicrobial drug resistance among bacteria by providing the selective pressure needed for the emergence of novel resistance determinants, selective pressure alone does not explain the increasing frequency with which *bla*_CTX-M_ alleles have been noted in bacterial populations in recent years (*16*,*33*). Although *bla*_CTX-M_ alleles tend to be located on transmissible plasmids and transposable elements, which clearly facilitate their dissemination, the repeated mobilization of the *bla*_CTX-M_s from the chromosomes principally among *Kluyera* species is most intriguing. The mechanistic basis underlying this repeated mobilization to plasmids remains unknown. Whether the chromosomes of *Kluyvera* species have some unique aspect that enhances the mobilization of the *bla*_CTX-M_ genes remains to be determined. Other factors, such as exposure of the isolates to specific antimicrobial agents or to environmental changes that facilitate the mobilization of *bla*_CTX-M_s to plasmids also need to be investigated.

Two insertion elements are known to contribute to the mobilization of *bla*_CTX-M_s. The first, which is associated with the CTX-M-2 and CTX-M-9 groups, is IS*CR1* (*34*) and the second, which is associated with the CTX-M-1, CTX-M-8, and CTX-M-25 groups is IS*Ecp1* (*35*). According to our phylogenetic analysis (Figure 1), 4 mobilizations can be attributed to each of these insertion type elements. Thus, both elements seem to promote equal frequencies of mobilization of *bla*_CTX-M_s. Notably, IS*CR1* was also reported to be responsible for the mobilization of both *bla*_VEB_ and *bla*_PER_ alleles, but neither of these resistance determinants has been reported to have an unusually high rate of mobilizations from chromosomal locations to plasmids.

Another factor that may contribute to the rate of mobilizations of the *bla*_CTX-M_ resistance determinants is the frequency of plasmids in bacterial populations. As the number of plasmids increases in microbial populations, so does the number of target replicons. A comparison of the percentage of bacterial strains that contained plasmids in the pre–antimicrobial drug era (*36*) with the percentage of contemporary strains that carry plasmids (*37**,**38*) indicates that the frequency of plasmid carriage has increased from 19% to 58%–100%, depending on the species surveyed. Although the collection methods and resistance detection assays varied in the studies used for this comparison (which may have introduced biases toward an increasing frequency of plasmids), few doubt that plasmid carriage is much more common among bacterial strains in the antimicrobial drug era (*39**,**40*). Unfortunately, specific information about plasmid carriage of *Kluyvera* species versus other *Enterobacteriaceae* is not available.

Regardless of the mechanism, the increased number of mobilizations of *bla*_CTX-M_s from their chromosomal locations among relatively rare human pathogens to plasmids that circulate widely among several important human and animal pathogens (particularly among *E. coli*) is a serious public health concern. The results of our study indicate the potential for an increase in the rate of mobilization of a variety of other resistance determinants to plasmids. Such an increase could result in more rapid mobilizations of novel resistance determinants and contribute to the accelerated spread of antimicrobial resistance determinants among a large spectrum of bacterial pathogens.
